# High altitude polycythemia and its maladaptive mechanisms: an updated review

**DOI:** 10.3389/fmed.2024.1448654

**Published:** 2024-08-27

**Authors:** Shijie Tang, Wenwen Zhou, Ling Chen, Hui Yan, Lei Chen, Fengming Luo

**Affiliations:** ^1^Department of High Altitude Medicine, Center for High Altitude Medicine, West China Hospital, Sichuan University, Chengdu, Sichuan, China; ^2^High Altitude Medicine Key Laboratory of Sichuan Province, West China Hospital, Sichuan University, Chengdu, Sichuan, China; ^3^Department of Pulmonary and Critical Care Medicine, West China Hospital, Sichuan University, Chengdu, Sichuan, China

**Keywords:** excessive erythrocytosis, high altitude polycythemia, hypoxemia, maladaptive mechanisms, review

## Abstract

High altitude polycythemia is a maladaptation of highlanders exposed to hypoxic environment, leading to high blood viscosity and severe cardiorespiratory dysfunction. Prolonged hypoxia causes respiratory depression and severe hypoxemia, and further mediates changes in genetic and molecular mechanisms that regulate erythropoiesis and apoptosis, ultimately resulting in excessive erythrocytosis (EE). This updated review investigated the maladaptive mechanisms of EE, including respiratory chemoreceptor passivation, sleep-related breathing disorders, sex hormones, iron metabolism, and hypoxia-related factors and pathways.

## 1 Introduction

Over 140 million people reside in highlands globally, among whom some people develop hypoxemia due to insufficient oxygen supply, leading to excessive erythrocytosis (EE) and increased blood viscosity, so-called high altitude polycythemia (HAPC), which is the most important contributor to chronic mountain sickness (CMS), a well-known maladaptive syndrome in highlands (1, 2).

Different populations show varied morbidity and adaptation. The incidence of EE was 2.39% in Lhasa, Tibet (3650 m) (3), whereas it increased to 4.5% among Peruvians in the Andes (3825 m) (4) and peaked at 44% in La Rinconada, Peru (5200 m) (5). Tibetans have been living in the Qinghai-Tibet Plateau for ten thousand years and successfully evolved with adaptive genetic mechanisms, thus showing a lower hematocrit (Hct) and incidence of EE than other highlanders living at the same altitude (6). Gene variations in EGLN1 and EPAS1 that regulated the pathways associated with hypoxia-inducible factor (HIF) were demonstrated to exert a significant influence on Tibetan adaptation (7, 8). Noticeably, distinct genetic adaptations to highlands were also revealed in other populations. However, the frequency of adaptive mutations such as the EPAS1 variant (rs570553380) was relatively low in Andean highlanders (9, 10), which resulted in a particular maladaptation to the hypoxic environment and more severe clinical manifestations (Table 1).

**TABLE 1:** Current genetic studies of highlanders globally.

Population	Gene	SNP	Adaptation/Disadaptation	Function	Source
Tibetan	EGLN1	rs186996510, rs12097901	A	Decreasing erythropoiesis	(7, 67)
	EPAS1	–	A	Decreasing anoxic response	(68)
	PPARA	–	A	Decreasing erythropoiesis	(68)
	ITGA6	rs3749148	A	–	(69)
	ERBB4	rs934607, rs141267844	A	–	(69)
		rs6710946	DA	–	(69)
	PIK3CD	–	DA	–	(70)
	COL4A3	–	DA	–	(70)
Andean	EPAS1	rs570553380	A	Decreasing hematocrit	(10)
	EGLN1	rs1769793 rs2064766, rs2437150, rs2491403, rs479200	A	Increasing VO_2_ max	(9)
	SENP1	–	DA	Increasing erythropoiesis	(71)
	ANP32D	–	DA	–	(71)
Han	EPAS1	rs75591953, rs75984373	A	–	(69)
	ITGA6	rs6744873	A	–	(69)
	ERBB4	rs17335043	A	–	(69)
Ladakhis	EGLN1, EPAS1, COQ7, NAPG, ADH6, DUOXA1	–	A	–	(72)
Ethiopian	CBARA1, VAV3, ARNT2, THRB, CIC, LIPE, PAFAHIB3	–	–	–	(73)

A, Adaptation; DA, Disadaptation; SNP, Single Nucleotide Polymorphism; –: unknown.

The maladaptive mechanisms that accelerate EE development are catching more attentions as the pathogenesis of EE has not been fully elucidated. Long-term hypoxic exposure stimulates the increase of erythrocyte count to enhance the transportation capacity of oxygen in blood, but promote the development of EE at the same time. In addition to the genetic adaptation, the maladaptive mechanisms of EE have been reported, including respiratory chemoreceptor passivation, sleep-related breathing disorders, sex hormones, iron metabolism, and hypoxia-related factors and pathways (11–17), which were reviewed with updates in this study, and prospects regarding new therapeutic strategies for HAPC were discussed as well.

## 2 Respiratory chemoreceptor passivation

Blunted chemoreceptor sensitivity means a decreased respiratory response to hypoxia, which has been observed in highlanders, leading to severe hypoxemia and EE. The chemoreflex drives of breathing were stimulated by sustained hypoxia to regulate ventilation response for the stabilization of arterial PO_2_ and were commonly evaluated by the hypoxic ventilatory response (HVR) and hypercapnic ventilatory response (HCVR) (18, 19).

Previous studies have documented that Han-Tibetan immigrants and Andeans with prolonged high-altitude living exhibited progressively weaker HVR and lower ventilation than Tibetans (20, 21). The discrepancy mainly accounted for a loss-of-function allele in Tibetans named EGLN1 that played a pivotal role in enhancing HVR (8). Additionally, Menuet et al. (22) also observed blunted HCVR in respiratory recordings of Tg6 mice with high erythropoietin (EPO) level in brain and plasma, and concluded that the high level of plasma EPO, but not cerebral EPO, acted on erythropoiesis and regulation of the blunted HCVR (22), possibly due to the reduced depletion of O2 and CO2, rather than the effect of EPO on CO2 chemosensitivity (23).

## 3 Sleep-related breathing disorders

Nocturnal periodic breathing (nPB) and nocturnal hypoxemia are frequent manifestations for sleep disorders in highlanders (12), which are attributed to insufficient ventilation and apnea caused by hypoxia in highlands (24). The nocturnal hypoxemia, represented by lower nocturnal blood oxygen saturation (SpO_2_), has been demonstrated to be relevant to higher Hct (25). Moreover, the apnea-hypopnea index (AHI), an indicator of nPB severity, was revealed to be negatively related to nocturnal SpO_2_, suggesting that more severe nBP correlated with lower nocturnal SpO_2_ (12). However, it is still doubtful whether nPB is only a clinical manifestation caused by nocturnal hypoxemia, since no univariate relevance between nPB and EE has been detected (26). In male Andeans, Hct was observed to be forecasted only by average sleep SpO_2_, AHI, and obstructive apnea index (OAI) (27), which indicated that the direct effect of nPB on Hct might be limited. Furthermore, a recent study suggested that ameliorating nPB did not improve the disordered sleep structure of highlanders, and thus more valuable indicators are needed to evaluate the relationship between sleep pattern and EE occurrence (24).

## 4 Sex hormones

The prevalence of HAPC is higher in men and postmenopausal women due to sex hormone regulation (4). Higher serum testosterone level was detected in male EE patients and up to 45% of postmenopausal women had high CMS scores with increased Hct in highlands, increased by 23% compared to premenopausal women (27–29). Azad et al. (17) collected samples from Andeans at an altitude of 4338m and established an induced pluripotent stem cell line to find that estrogen significantly decreased hypoxia-induced erythropoiesis in a dose-dependent manner (17). The estrogen directly reduced the expression level of GATA1, a transcription factor regulating erythroid differentiation, and inhibited its downstream targets, including Alas2, Bcl-xL, and erythropoietin receptor (EpoR), to decrease hypoxia-induced erythropoiesis and increase erythrocyte apoptosis (17). Moreover, the mRNA expressions of GATA1, vascular endothelial growth factor (VEGF), and HIF-1 were distinctly restrained by estrogen beta signaling that was identified as a dominant pathway involved in inhibiting EE progression (17).

## 5 Iron metabolism

Iron is an important component of hemoglobin and is involved in EE occurrence. EE patients among Han immigrants living in the Qinghai-Tibet Plateau showed higher concentrations of serum iron, Hct, serum soluble transferrin receptor, and serum ferritin than healthy highlanders, indicating increased iron availability and reserves (30). The increased expression of HIF-2α in the intestine of EE mice upregulated the hub gene expression related to iron metabolism, such as Dmt1, Dcytb, Fpn, Tfrc, and Fth, to enhance iron availability and thus promote erythropoiesis (13). Hepcidin, a peptide hormone, plays a crucial role in iron homeostasis by inhibiting excessive iron mobilization. In the hypoxic environment, the hepcidin expression level was decreased, followed by stored iron releasing and consequent erythropoiesis, which was involved in downregulation of STAT3 signaling pathway (30). Moreover, red pulp macrophages (RPMs) in the spleen serve as a cleaner for the removal of abnormal erythrocytes, and the ferroptosis of RPMs due to lipid peroxidation was observed in C58BL/6 mice with hypoxia, resulting in decreased RPMs and erythrocytic clearance (31). Besides, in a recent study, men living in the highest cities of the world indicated that their iron stores remained stable regardless of whether they suffered from CMS, which implied the highlanders might have a unique mechanism for maintaining an efficient balance between iron absorption/storage and its consumption (32).

## 6 Hypoxia-related factors and pathways

### 6.1 HIF-EPO pathway and VHL

EPO, mainly generated by the kidneys, is induced in anoxic environment and binds to EpoR, then activating its downstream signals to further promote erythropoiesis and reduce erythrocyte apoptosis (33). HIF is a regulator of EPO, including HIF-1α and HIF-2α, and involved in acute and chronic hypoxia respectively, which mediates erythrocyte formation by affecting the transcription and synthesis of EPO (34). Recent studies have suggested that HIF-2α/EPO-related pathway plays a dominant role in hypoxia-induced erythrocytosis (35).

VHL is a tumor suppressor involved in the ubiquitylation and degradation of HIFs (36, 37). Hypoxia promoted methylation of the VHL promoter by increasing the expression of DNA methyltransferase, DNMT3α and DNMT3β, leading to inhibition of VHL expression (14, 38). The downregulated VHL protected the HIF-2α from degradation and contributed to erythropoiesis (14). Moreover, the mRNA expression of HIF-2α, but not HIF-1α, was upregulated in the bone marrow cells of patients with EE (14). Overall, hypoxia inhibits VHL to reduce the degradation of HIFs and promotes HIF-2α/EPO-related pathway, resulting in EE.

### 6.2 EPO/soluble EpoR ratio

EPO regulates erythropoiesis through the EPO/EpoR pathway (39), and soluble EpoR (sEpoR) is a protein produced by alternative splicing of EpoR mRNA and can reduce EPO availability by competitively combining with EPO (40). Noticeably, only part of EE patients exhibited raised EPO levels, while other patients had normal EPO levels, suggesting, in addition to EPO, sEpoR may play a role in pathogenesis of EE (41). Hypoxia-induced downregulation of sEpoR was observed to modulate respiratory status and increase oxygen transport in mice (42). Further, sEpoR was decreased in both normal and high EPO groups of Andeans with EE at an altitude of 4340m while EPO/sEpoR was positively correlated with Hct and negatively correlated with SpO_2_ (41). Another study reported a higher EPO/sEpoR ratio during the night and early morning in Andean highlanders, despite EPO concentration showing no obvious change over time (43). The proportion of lower nocturnal SpO_2_ ( ≤ 80%) was higher in CMS patients, leading to a decrease in sEpoR and an increase in nocturnal EPO/sEpoR ratio and erythropoiesis (43). Overall, the EPO/sEpoR ratio rather than EPO may play a key role in EE.

### 6.3 SENP1-HIF/GATA1 pathway

SENP1 is a nuclear small ubiquitin-related modifier protein (SUMO) protease. Previous studies demonstrated that SENP1 was involved in the pathogenesis of CMS and regulated hypoxia-stimulating erythropoiesis (44). A decrease in the number of erythroid colony-forming units (CFU-e) was observed in SENP1 knockout mice due to EPO downregulation and increased apoptosis of erythroid progenitor cells (45). SENP1 regulated EPO production and erythrocyte metabolism through HIF and GATA-1 in the hypoxic environment (15, 45). HIF is sumoylated intracellularly and then degraded by SUMO-targeted ubiquitin ligases. SENP1 protects sumoylated HIF from degradation by desumoylation and increases the downstream effects of HIF under hypoxic conditions (45, 46), then further regulates the production of EPO and erythrocytes. GATA1, a transcription factor that regulates erythroid differentiation, interacts with a variety of hematopoietic transcription factors and is involved in the regulation of erythropoiesis genes such as heme biosynthetic enzymes, hemoglobin, EpoR, and anti-apoptotic protein (Bcl-xL) (47–51), and GATA1-deficient mice exhibited severe anemia and eventual death (48). SENP1 can upregulate the expression of GATA1 and its downstream factor Bcl-xL during hypoxia, then contributing to erythrocyte proliferation and apoptosis inhibition (15, 48). Overall, under hypoxic environment, SENP1 upregulates the expression of HIF and GATA1 to increase EPO and erythropoiesis.

### 6.4 HIKER/LINC02228-CSNK2B-GATA1

long-chain noncoding RNAs (lncRNAs) are transcripts that can regulate gene transcription, having a vital role in cardiovascular, neurological, endocrine systems, cancer occurrence, and so on (52), which was recently revealed to be correlated with EE (53). An RNA-Seq analysis was conducted to investigate the effect of gene transcription of erythroid cells on EE patients and significant differences in the expression of lncRNAs were discovered between EE and non-EE subjects (53). Among these, the lncRNA showing the most notable change was named hypoxia-induced kinase-mediated erythropoietic regulator (HIKER)/LINC02228, which was upregulated in vitro with EE phenotype and proved to be significant for regulating erythroid progenitors under hypoxic conditions by increasing the expression of casein kinase 2β unit (CSNK2B) (53), a downstream factor of HIKER/LINC02228. CSNK2B was suggested to promote erythrocyte growth and upregulation of GATA1 (54), and CSNK2B knockout mice showed severe anemia (53). Overall, hypoxia upregulates the HIKER/LINC02228 in EE patients to increase CSNK2B expression, which further promotes the GATA1 level and thus erythrocyte growth.

## 7 Discussion

This updated review suggested that the pathogenesis of HAPC was involved in not only lack of the adaptive genetic background, but also complicated maladaptive mechanisms that were discussed above. However, the advances in HAPC treatment are slow. In addition to recommendation of migration to low-altitude areas, the conventional treatments for HAPC include phlebotomy and hemodilution (55, 56), which often result in hypovolemia, iron deficiency and even exacerbation of pulmonary hypertension (57), but recently, erythrocytapheresis has been widely used to reduce Hct in HAPC by seperating erythrocytes from the circulation with hemodynamic stability (58).

However, targeting on the maladaptive mechanisms could be more effective for treatment of HAPC. Six-month administration of acetazolamide, a carbonic anhydrase inhibitor, stimulated respiratory response and rectify hypoxemia, and thus reduced Hct, EPO and pulmonary vascular resistance and even improved sleep-related breathing disorders (59, 60). Noticeably, as a methylated lipophilic analog of acetazolamide, methazolamide was shown to reduce Hct and blood viscosity dose-dependently in a rat model of EE, but with fewer side effects than acetazolamide, indicating a promising prospect for methazolamide in HAPC treatment (61, 62).

Maca is a plant growing in Peru, and long-term use of maca reduced the Hct in EE patients (63), which was implicated in regulation of sex hormone and its related pathways (64). Likewise, as a traditional Chinese medicinal plant, astragalus membranaceus, decreased erythropoiesis by inhibiting the differentiation of hematopoietic stem cells into erythroid lineage and downregulating the expression of HIF-1a, EPO and GATA-1 in HAPC mice (65).

Interestingly, it was demonstrated that arginine could inhibit the development of EE by reducing EPO/EpoR via upregulation of miR-144-5p in a rat model of CMS (66). Moreover, HIF-2α antagonists, CSNK2B inhibitors and DNMT inhibitors were reported to improve hypoxia-induced EE in experimental studies, suggesting potential pharmaceutical values (13, 14, 53).

Taken together, although the treatment of HAPC is dependent on the conventional methods, new strategies targeting on the maladaptive mechanisms may lead to better therapeutic effects in the forthcoming future.
